# A comparative study of discriminating human heart failure etiology using gene expression profiles

**DOI:** 10.1186/1471-2105-6-205

**Published:** 2005-08-24

**Authors:** Xiaohong Huang, Wei Pan, Suzanne Grindle, Xinqiang Han, Yingjie Chen, Soon J Park, Leslie W Miller, Jennifer Hall

**Affiliations:** 1 Division of Biostatistics, School of Public Health, University of Minnesota, Minneapolis, MN 55455, USA; 2 Cardiovascular Division, Department of Medicine, Medical School, University of Minnesota, Minneapolis, MN 55455, USA

## Abstract

**Background:**

Human heart failure is a complex disease that manifests from multiple genetic and environmental factors. Although ischemic and non-ischemic heart disease present clinically with many similar decreases in ventricular function, emerging work suggests that they are distinct diseases with different responses to therapy. The ability to distinguish between ischemic and non-ischemic heart failure may be essential to guide appropriate therapy and determine prognosis for successful treatment. In this paper we consider discriminating the etiologies of heart failure using gene expression libraries from two separate institutions.

**Results:**

We apply five new statistical methods, including partial least squares, penalized partial least squares, LASSO, nearest shrunken centroids and random forest, to two real datasets and compare their performance for multiclass classification. It is found that the five statistical methods perform similarly on each of the two datasets: it is difficult to correctly distinguish the etiologies of heart failure in one dataset whereas it is easy for the other one. In a simulation study, it is confirmed that the five methods tend to have close performance, though the random forest seems to have a slight edge.

**Conclusions:**

For some gene expression data, several recently developed discriminant methods may perform similarly. More importantly, one must remain cautious when assessing the discriminating performance using gene expression profiles based on a small dataset; our analysis suggests the importance of utilizing multiple or larger datasets.

## Background

Human heart failure is a complex disease diagnosed in over 500,000 American people every year, causing more than 250,000 deaths annually. It may arise from coronary atherosclerosis, exposure to toxins, infection, inflammation, valvular disease leading to volume/pressure overload, or an underlying genetic or idiopathic event [1-3]. Emerging work suggests the heterogeneity of heart failure. For example, patients with ischemic heart failure have decreased survival compared to the non-ischemic heart failure [4,5] and respond differently to therapies [6-9]. Although benefits can be achieved using ischemic heart failure therapy for idiopathic heart failure (and vice versa), cure rates will be markedly diminished, and unwarranted toxicities problems will be encountered. It may be critical to distinguish these characteristically similar but clinically somewhat distinct heart failures, to better optimize therapy. The ability to distinguish between different etiologies of heart failure may be essential to guide appropriate therapy and determine prognosis for successful treatment.

A new approach to discriminating etiologies of heart failure is gene expression profiling using DNA microarray technology, which has been shown to be promising in the diagnosis of human diseases or subdiseases, especially in cancer [10-12]. Recent genomic studies by three separate groups have demonstrated a distinct etiology dependent genomic pattern in the failing heart [13-16]. These studies offer hope that the microarray gene expression analysis could potentially add to conventional laboratory approaches to diagnose different underlying etiologies of heart failure while simultaneously enhance prognostic criteria. It was hypothesized that heart failure arising from different underlying etiologies present with different gene expression patterns and that these differences could be used as a diagnostic tool. Here we test the hypothesis with two human heart failure datasets from different institutions.

Sample classification with gene expression data is statistically challenging due to the "small *n*, large *p*" problem [17]: the number of samples *n *is much smaller than the number of genes or predictors *p*. In our first dataset, we have *n *= 30 and *p *> 20000. Many new statistical methods have been developed or adapted to face the challenge. With more and more new methods emerging and existing methods being adapted, it becomes increasingly compelling for practitioners to compare and assess their performance, but there are few such comparative studies [18-20]. Huang and Pan [19] compared several methods, including partial least squares (PLS) [21], nearest shrunken centroid (SC) [12], and a penalized PLS (PPLS), for binary classification of gene expression data. They found that these methods are competitive. More recently, some authors [22,20] have shown the promising performance of least absolute shrinkage and selection operator (LASSO) [23] and random forest (RF) [24]. It is our main goal to evaluate and compare these methods using two human heart failure datasets. For this purpose, we also extend the PPLS, originally proposed for binary classification, to multiclass classification. We found that the above five statistical methods perform similarly. Furthermore, our analysis stresses the importance of utilizing multiple datasets for classification purposes.

## Results

### Minnesota data

Myocardial tissue samples from the left ventricular apex of patients with severe refractory heart failure were collected at the time of the left ventricular assist device (LVAD) placement at the University of Minnesota Medical School. A total of 30 tissue samples were processed for microarray analysis on the Affymetrix Human Genome U133A chip containing ~22,000 genes. The initial data analysis was completed using Affymetrix Microarray Suite (MAS 5.0). A more complete description on the data is provided in [25].

The heart failure patients are divided into three classes according to the underlying etiology. Patients with clinical ECHO and EKG evidence, history of previous myocardial infarctions, and direct observation of the heart for confirmation of infarction at the time of LVAD implantation are defined as ischemic. Patients with an ischemic etiology were further divided into two classes: patients with ischemia but without acute myocardial infarctions (ischemic class) and patients with ischemia that have had an acute myocardial infarctions within ten days of LVAD implant (IM class), the remaining patients were assigned to the idiopathic class. Among the total 30 samples, 10 of them are ischemic, 7 are IM and 13 are idiopathic.

### PGA data

The PGA data were obtained in another heart failure study conducted at the Cardio-Genomics PGA (Programs for Genomic Applications) at the Harvard Medical School. Myocardial samples were collected from patients undergoing heart transplantation whose failure arises from different etiologies (e.g. idiopathic, ischemic, alcoholic, valvular, and hypertrophic) and from normal organ donors whose hearts were not used for transplants. The transcriptional profile of the mRNA in these samples was also measured with Affymetrix oligonucleatide microarray technology. HG-U133 plus 2 chips containing 54,675 probe sets were used and data were analyzed in MAS 5.0. In the PGA data set there were 11 normal samples, 11 ischemic samples and 14 idiopathic samples. The PGA dataset is publicly available at Genomics of Cardiovascular Development, Adaptation, and Remodelling. NHLBI Program for Genomic Applications, Harvard Medical School with URL: .

In order to make the results comparable to those based on the HG-U133A chips used in the Minnesota data, we matched the probe sets on a HG-U133 plus 2 chip with those on a HG-U133A chip. Only six probe sets on the HG-U133 plus 2 chip could not be found on a HG-U133A chip. Hence we used the remaining 22277 ( = 22283 - 6) probe sets in the following analyses with the PGA data.

### Classification with Minnesota data: *One-against-others *approach

Table 1 reports the LOOCV misclassification errors of the five classification methods for the Minnesota data with models starting from different numbers of top ranked genes ranging from 50 to all genes. Four kinds of errors are reported for each method: three *one-against-others *two-class LOOCV misclassification errors (Ischemic vs. others, IM vs. others and Idiopathic vs. others) and one three-class LOOCV misclassification error. Note that throughout this article, three-class classification results for SC and RF were obtained by direct applications of SC and RF, rather than by combining multiple binary classifications.

**Table 1:** LOOCV errors for three-class classification with Minnesota data: all patients.

# of top genes	Isch vs other					IM vs other					Idio vs other					Overall				
	
	PPLS	PLS	SC	LSO	RF	PPLS	PLS	SC	LSO	RF	PPLS	PLS	SC	LSO	RF	PPLS	PLS	SC	LSO	RF
50	7	9	8	11	12	6	8	6	7	9	8	6	5	8	6	14	11	11	12	14
100	9	6	8	9	11	6	7	6	7	8	10	9	5	11	8	11	9	11	13	13
200	9	7	12	9	11	7	9	7	7	9	6	7	5	6	7	12	11	12	8	14
400	11	11	13	10	12	8	9	8	7	8	10	8	8	8	8	16	15	14	11	15
800	8	11	12	13	12	9	9	8	8	8	8	8	7	10	8	15	17	15	14	16
1600	12	12	11	11	11	9	8	8	5	8	12	8	5	7	7	16	17	13	11	13
3200	10	12	9	15	10	9	9	7	6	7	9	7	5	10	8	14	14	14	14	13
6400	11	11	10	15	10	9	10	9	8	7	9	8	5	8	8	15	14	13	15	12
9600	10	11	10	15	10	10	8	7	9	7	7	8	4	9	10	14	12	15	16	15
12800	13	12	11	15	11	7	7	7	9	7	8	8	5	10	9	15	13	15	17	17
16000	13	12	13	15	10	8	7	10	9	7	8	8	5	10	9	15	13	14	17	16
19200	13	12	13	15	11	9	7	10	9	7	8	8	5	10	8	15	14	14	17	17
22283	13	13	11	15	10	7	6	11	9	7	7	7	5	10	10	14	14	14	18	16

Based on Table 1, we first note that the performances of any classifier is sensitive to the number of top ranked genes one starts with. For example, in the three-class classification, LASSO made 8 errors when only the top 200 genes are considered but made 18 errors when all 22283 genes are used. But there is no obvious relationship between the gene subset size and the five methods' performances.

In the two-class classification problem of ischemic vs. others, PPLS, PLS, SC, LASSO and RF yielded LOOCV errors range from 7 to 15, 6 to 13, 8 to 13, 9 to 15 and 10 to 12 respectively. The five methods obtained similar numbers of errors in all instances. In the two-class classification problems of IM vs. others and idiopathic vs. others, all five methods yielded similar numbers of errors. In the overall three-class classification, again all the five classification methods perform similarly. There is no clear evidence that one method is clearly superior to others.

In order to assess whether a gene expression profile is affected by gender, we classified the 23 samples from male patients only. Among the 23 samples for male patients, 9 of them are ischemic, 7 are IM and 7 are idiopathic.

Table 2 reports the LOOCV misclassification errors of these five methods for the Minnesota data with males only. Again, the models start from different numbers of top ranked genes. The three two-class LOOCV misclassification errors and one three-class LOOCV misclassification error are estimated for each method as described before.

**Table 2:** LOOCV errors for three-class classification with Minnesota data: males only.

# of top genes	Isch vs other					IM vs other					Idio vs other					Overall				
	
	PPLS	PLS	SC	LSO	RF	PPLS	PLS	SC	LSO	RF	PPLS	PLS	SC	LSO	RF	PPLS	PLS	SC	LSO	RF
50	11	9	11	10	12	7	6	5	7	7	5	6	6	6	5	15	12	11	14	15
100	10	10	10	11	11	5	7	5	8	7	5	6	6	5	8	10	12	11	13	14
200	12	12	16	10	12	6	7	6	8	5	6	6	7	7	8	13	12	11	13	15
400	10	12	15	10	12	6	8	7	7	7	5	7	8	6	9	14	14	11	14	13
800	14	12	13	9	12	6	7	6	8	7	5	6	8	6	9	12	11	12	14	15
1600	15	15	12	7	10	6	4	7	8	6	5	5	8	8	8	13	12	13	13	17
3200	11	11	11	10	11	7	7	7	8	8	5	5	5	8	8	14	11	14	15	15
6400	11	12	13	10	11	6	8	6	11	7	7	6	4	8	8	11	14	15	19	14
9600	9	11	14	9	10	7	7	6	8	7	8	7	4	8	8	13	13	13	19	15
12800	9	10	15	10	10	5	8	5	8	8	8	8	5	8	7	12	14	15	20	15
16000	13	12	15	10	9	8	6	5	8	7	9	7	5	8	7	14	12	14	20	17
19200	13	10	11	10	9	6	6	5	8	7	7	6	6	8	8	14	12	13	20	17
22283	12	11	9	10	11	7	5	5	8	8	5	5	6	8	8	15	11	14	20	15

Based on Table 2, again we note that the classification performances of PPLS, PLS, SC, LASSO and RF can be quite sensitive to the number of top ranked genes one uses. For example, in the *one-against-others *two-class classification problem of ischemic vs. others, PPLS made 10 errors when the top 400 genes are considered but the number of errors suddenly increases to 14 when the top 800 genes are used. Again there is no obvious relationship between the gene subset size and the five methods' performances.

Comparing PPLS, PLS, SC, LASSO and RF, we find that the five methods perform very similarly in almost all instances. There is no evidence that any method is clearly the best. One thing we noticed about LASSO is that when the model contains many genes, say top 12800, then it gives more errors than PPLS, PLS, SC and RF in the three-class classification. This could happen by chance since we did not observe the same trend in the decomposed *one-against-others *binary classifications.

As in Table 1, the results in Table 2 suggest that discriminating ischemic group from the other two groups was less accurate than distinguishing IM from the other two groups and separating idiopathic group from the other two groups.

If we compare Table 1 (30 patients) with Table 2 (23 male patients), we can see that the misclassification error rates of all the five methods in Table 2 are much higher than those in Table 1. Reduced sample size is likely a factor.

To see whether the high prediction errors are due to the presence of the three classes, we considered a binary classification problem. We applied all the five methods to the 10 ischemic and 13 idiopathic samples. We also assessed the classification accuracy on the male patients with 9 ischemic and 7 idiopathic samples. The classification results were shown in Table 3.

**Table 3:** LOOCV errors for two-class classification with Minnesota data: ischemic vs idio-pathic.

# of top genes	All (23 samples)					Males (16 samples)				
	
	PPLS	PLS	SC	LASSO	RF	PPLS	PLS	SC	LASSO	RF
50	10	6	5	6	6	10	9	11	8	11
100	7	7	5	7	6	10	9	11	11	12
200	9	9	7	11	6	9	9	11	11	9
400	6	8	7	11	8	9	9	8	12	9
800	6	9	8	4	7	10	10	9	12	10
1600	5	8	8	8	8	10	10	9	12	10
3200	9	10	9	8	10	8	8	10	12	9
6400	7	7	9	8	9	6	6	10	12	9
9600	9	8	8	7	8	6	6	10	12	8
12800	8	8	8	8	11	6	6	10	12	10
16000	8	7	7	8	9	7	6	10	12	11
19200	11	8	6	7	11	8	7	9	12	9
22283	9	10	5	7	9	8	8	9	12	7

From Table 3, we can see that all the five methods have very similar performances in classifying ischemic and idiopathic samples. If we compare the classification performances of these five methods with/without female patients, taking the sample size into consideration, we can see that the misclassification error rates with only male patients is much higher than those with all patients. This again is probably because the sample size (16) with only males is smaller. In particular, we noticed that LASSO is more sensitive to the small sample size.

### Other models

In the previous classification problems, we only included linear terms of gene expression levels in a model. We also considered expanded models including squared terms of each gene's expression levels. The motivation is to possibly improve model fitting, for instance, to avoid masking in linear models [26]. In this way, the number of variables in the new data is doubled (the original variables plus their squared terms) and we have 44566 variables. We repeated all the previous classification procedures and found that the classification performance did not improve (results not shown).

### Classification with Minnesota data: *pair-wise *approach

We repeated the three-class classification with PPLS via the *pair-wise *approach. The results are included in Table 4. We assessed the classification of PPLS by including all 30 patients and with 23 male only. By comparing Table 4 to Table 1 – 2, we can see that the PPLS via *one-against-others *approach gives much smaller errors. This suggests that for this specific problem, the *one-against-others *approach is probably better. Again, we see the classification with male patients gives much larger LOOCV misclassification error rates.

**Table 4:** LOOCV errors for three-class classification: PPLS with *pair-wise *approach.

#of top genes	Minnesota data	
	
	All patients	Male
50	18	16
100	13	18
200	16	18
400	17	17
800	18	14
1600	17	16
3200	16	14
6400	15	15
9600	17	15
12800	17	14
16000	19	14
19200	14	16
22283	19	16

### Classification with PGA data: *one-against-others *approach

Table 5 reports the LOOCV misclassification errors of the five methods for the PGA data. Four kinds of errors are reported for each method: three *one-against-others *two-class LOOCV misclassification errors (Normal vs. others, Ischemic vs. others, and Idiopathic vs. others) and one three-class LOOCV misclassification error.

**Table 5:** LOOCV errors for three-class classification with PGA data: all patients.

# of top genes	Normal vs other					Isch vs other					Idio vs other					Overall				
	
	PPLS	PLS	sc	LSO	RF	PPLS	PLS	SC	LSO	RF	PPLS	PLS	SC	LSO	RF	PPLS	PLS	SC	LSO	RF
50	0	0	0	0	0	3	1	2	2	1	5	1	5	1	2	2	1	1	1	1
100	0	0	0	0	0	4	2	3	1	2	3	1	3	1	1	1	1	1	1	1
200	0	0	0	1	0	5	1	2	1	2	2	2	2	2	1	1	1	1	2	1
400	0	0	0	0	0	3	1	2	4	2	2	1	2	2	2	2	1	1	2	1
800	0	0	0	0	0	3	1	2	1	2	2	1	2	2	2	2	1	1	1	1
1600	0	0	1	0	0	2	1	2	1	2	2	2	2	2	2	2	1	1	2	1
3200	0	0	2	0	0	3	2	2	2	2	2	3	3	2	2	2	2	1	2	1
6400	0	0	2	0	0	3	2	2	1	2	2	2	3	2	2	2	2	2	2	1
9600	0	0	0	0	0	2	1	2	1	2	3	2	2	2	2	2	1	1	2	1
12800	0	0	0	0	0	3	1	2	1	2	3	2	2	2	2	1	1	1	2	1
16000	0	0	0	0	0	2	1	2	1	2	3	2	2	2	2	1	1	1	2	1
19200	0	0	0	0	0	2	1	2	1	2	2	1	2	2	2	1	1	1	2	1
22277	0	0	0	0	0	2	1	2	1	2	2	1	2	2	2	1	1	1	2	1

Based on Table 5, we can see that the classification performances of PPLS, PLS, SC, LASSO and RF are quite stable with different numbers of top ranked genes one uses. In the two-class classification problem of normal vs. others, the five classification methods almost perform perfectly, where PPLS, PLS and RF have 0 errors in all circumstances, SC has 0 errors in all situations except for 3 cases and LASSO has 1 error in one case and 0 errors in all other cases. In the two-class classification problems of ischemic vs. others and idiopathic vs. others, PPLS, PLS, SC, LASSO and RF yielded 1–5 errors (mostly with 1–3 errors). That the problem of distinguishing normal from the others is the easiest confirms that the normal class is more separable from the other two classes. As for the three-class classification, the errors range from 1 to 2 and it is almost perfect.

It may be argued that a classification involving normal samples should be much easier because the normal class is very different from the other two classes. Correct diagnosis between ischemic and idiopathic would be much more challenging. Hence we conducted a two-class classification with 11 ischemic and 14 idiopathic samples. The results are shown in Table 6. The five methods perform almost perfectly: the misclassification errors range from 1 to 3 for all five methods in all models.

**Table 6:** LOOCV errors for two-class classification with PGA data: ischemic vs idiopathic.

# of top genes	PPLS	PLS	SC	LASSO	RF
50	3	2	2	2	2
100	1	1	2	1	1
200	1	1	2	1	2
400	1	1	1	2	1
800	1	1	1	3	1
1600	1	1	1	2	1
3200	1	2	1	1	1
6400	1	3	1	1	1
9600	1	3	1	1	1
12800	1	3	1	1	1
16000	1	2	1	1	1
19200	1	1	1	1	1
22277	1	1	1	1	1

### Genes identified

We consider genes remaining in a final model for each method. To save space, we restrict attention to models starting with all the genes for binary discrimination between ischemic and idiopathic samples. Briefly, LOOCV was first used to select any tuning parameters in a method (e.g. number of components in a PLS model), then a model with the selected parameters was fitted using all the samples. Except that all the genes are used in a final PLS model, for any of the other methods there may be fewer genes remaining in the final model. In particular, LASSO can select at most *n *genes with *n *the number of the samples. It turned out that random forest also used many of the genes.

Tables 7 and 8 lists the genes selected by at least four or three methods for the Minnesota data and PGA data respectively. It can be seen that there is no overlap at all between the two gene lists. Although the same genes are not identified from the two datasets, it is clear that the beta-adrenergic signalling pathway is likely a discriminatory pathway, given the inclusion of CREM in the Minnesota data and AKAP6 in the PGA data. Furthermore, the inclusion of metabolic-related genes, such as ATPase and GAPD, is not surprising given the class of ischemic tissue.

**Table 7:** Genes selected by ≥ 4 methods in two-class classification (ischemic vs idiopathic) with Minnesota data. The last row gives the total numbers of the genes in the final models.

Probe Set	Gene	Rank	PPLS	PLS	SC	LSO	RF
215066_at	PTPRF: protein tyrosine phosphatase, receptor type, F	1	X	X		X	X
212008_at	UBXD2: UBX domain containing	2	X	X	X		X
217234_s_at	VIL2: villin 2 (ezrin)	3	X	X	X		X
202092_s_at	BART1: binder of Arl Two	5	X	X	X		X
218318_s_at	NLK: nemo-like kinase	7	X	X		X	X
212062_at	ATP9A: ATPase, Class II, type 9A	9	X	X	X		X
218208_at	FLJ22378: hypothetical protein FLJ22378	13	X	X	X		X
212093_s_at	MTSG1: mitochondrial tumor suppressor gene 1	14	X	X	X		X
214543_x_at	QKI: quaking homolog, KH domain RNA binding (mouse)	24	X	X	X		X
209487_at	RBPMS: RNA binding protein with multiple splicing	26	X	X	X	X	X
202877_s_at	C1QR1: complement component 1, q subcomponent, receptor 1	31	X	X		X	X
64438_at	FLJ22222: hypothetical protein FLJ22222	40	X	X		X	X
221928_at	LOC283445: hypothetical protein LOC283445	44	X	X	X		X
212556_at	SCRIB: scribble	48	X	X		X	X
202641_at	ARL3: ADP-ribosylation factor-like 3	50		X	X	X	X
201559_s_at	CLIC4: chloride intracellular channel 4	64	X	X	X		X
216231_s_at	Homo sapiens transcribed sequence with strong similarity to protein pdb:3HLA (H.sapiens) B Chain B, Human Class I Histocompatibility Antigen A2.1 (HLA-A2.1 Human Leucocyte Antigen)	73	X	X	X	X	X
220477_s_at	C20orf30: chromosome 20 open reading frame 30	76	X	X	X		X
208879_x_at	C20orfl4: chromosome 20 open reading frame 14	88	X	X		X	X
207630_s_at	CREM: cAMP responsive element modulator	107	X	X		X	X
212117_at	ARHQ: ras homolog gene family, member Q	116	X	X	X		X
212904_at	KIAA1185: KIAA1185 protein	121		X	X	X	X
M33197_5_at	GAPD: glyceraldehyde-3-phosphate dehydrogenase	161	X	X	X		X
213507_s_at	KPNB1: karyopherin (importin) beta 1	163	X	X	X		X
207627_s_at	TFCP2: transcription factor CP2	208	X	X		X	X

Total	-	-	275	22283	36	22	883

**Table 8:** Genes selected by ≥ 3 methods in two-class classification (ischemic vs idiopathic) with PGA data, The last row gives the total numbers of the genes in the final models.

Probe Set	Gene	Rank	PPLS	PLS	SC	LSO	RF
206375_s_at	HSPB3: heat shock 27kDa protein 3	1	X	X		X	X
202430_s_at	PLSCR1: phospholipid scramblase 1	2	X	X		X	X
209948_at	KCNMB1: potassium large conductance calcium-activated channel, subfamily M, beta member 1	3	X	X	X		
AFFX-TrpnX-5_at		4	X	X	X		X
212929_s_at	KIAA0592: KIAA0592 protein	6	X	X	X		X
221415_s_at	MYCBP: c-myc binding protein	7	X	X	X		
219099_at	C12orf5: chromosome 12 open reading frame 5	8	X	X		X	
219383_at	FLJ14213: hypothetical protein FLJ14213	10	X	X	X		X
208846_s_at	VDAC3: voltage-dependent anion channel 3	11	X	X	X		X
205359_at	AKAP6: A kinase (PRKA) anchor protein 6	12	X	X			X
202324_s_at	GOCAP1: golgi complex associated protein 1, 60 kDa	14	X	X		X	X
208736_at	ARPC3: actin related protein 2/3 complex, subunit 3, 21 kDa	15	X	X			X
215700_x_at	CPNE6: copine VI (neuronal)	16	X	X	X		
207600_at	KCNC3: potassium voltage-gated channel, Shaw-related subfamily, member 3	17	X	X			X
217386_at		18	X	X		X	
200961_at	SPS2: selenophosphate synthetase 2	19	X	X		X	X
211476_at	MYOZ2: myozenin 2	21	X	X			X
209682_at	CBLB: Cas-Br-M (murine) ecotropic retroviral transforming sequence b	22	X	X		X	
208769_at	EIF4EBP2: eukaryotic translation initiation factor 4E binding protein 2	23	X	X			X
208162_s_at	FLJ10232: hypothetical protein FLJ10232	25	X	X			X
210500_at	NICE-4: NICE-4 protein	26	X	X			X
216721_at	LOC253512: hypothetical protein LOC253512	27	X	X			X
206475_x_at	CSH1: chorionic somatomammotropin hormone 1 (placental lactogen)	28	X	X			X
210561_s_at	WSB1: SOCS box-containing WD protein SWiP-1	29	X	X			X
206598_at	INS: insulin	30	X	X			X
219293_s_at	PTD004: hypothetical protein PTD004	75		X		X	X
207431_s_at	DEGS: degenerative spermatocyte homolog, lipid desaturase (Drosophila)	139		X		X	X
205207_at	IL6: interleukin 6 (interferon, beta 2)	272		X		X	X
221775_x_at	RPL22: ribosomal protein L22	1043		X		X	X
200897_s_at	KIAA0992: palladin	1175		X		X	X

total	-	-	32	22277	7	22	548

We also give the univariate ranks of the genes (based on the F-statistics for the two classes) in the above two tables. It shows that the two sets of genes (or more generally, the genes in a final model) may not include some genes ranked high in the univariate ranking while including some ranked low, highlighting a possible limitation of solely depending on univariate ranks to select important genes.

### More numerical results

#### PLS plots

**Figure 1:**
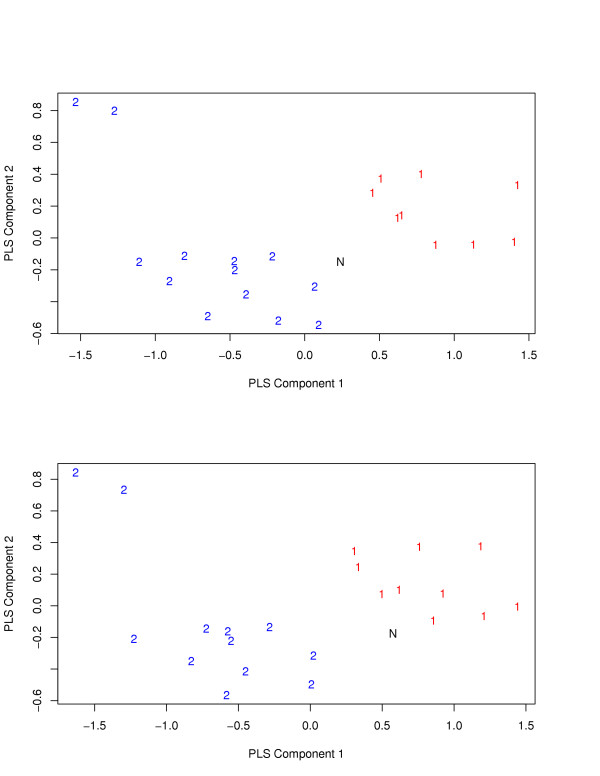
PLS plots for two cases in LOOCV for the Minnesota data comparing ischemic vs idiopathic. In both cases, the new sample labeled as "N" (i.e. left-out sample in LOOCV), belonging to class 1 and 2 respectively in the top and the bottom panels, is closer to the other group different from its true class, leading to misclassifications.

To further explore why the methods all work much better for the PGA data than for the Minnesota data, we drew some plots using the first and the second PLS components for binary classification of ischemic vs idiopathic groups. We found that, for both datasets, there was a clear separation between the two groups. However, in LOOCV, although again the two groups were separable for both datasets, the left-out sample was more likely to be closer to the other group than to its true group for the MInnesota data, leading to a high LOOCV error rate; Figure 1 gives two examples. In contrast, in the PGA data, a left-out sample tended to be close to its true group, resulting in a low LOOCV error rate. For more details see Supplemental Materials.

#### Permutation tests

Because of high misclassification error rates with the Minnesota data, it is of interest to investigate whether there is any signal at all in the data. This can be accomplished by a permutation test that compares misclassification errors resulting from using the original data with that from randomly permuted data; a *P*-value is defined as the proportion of permuted datasets with misclassification errors fewer than that of the original data. To generate a randomly permuted data, we randomly permute the class labels of the original data. Because all the methods have similar performance, we consider the nearest shrunken centroid method with the Minnesota data. Tables 9 and 10 summarize the results of misclassification errors for 50 randomly permuted datasets for three- and two-class classifications respectively. It can be seen that the misclassification errors based on the original data are fewer than that based on the permuted data, leading to small *P*-values. This implies that, although there are relatively high misclassification error rates with the Minnesota data, the methods perform significantly better than a random guess.

**Table 9:** LOOCV three-class misclassification errors with the original Minnesota data and the percentiles of LOOCV errors with 50 permuted datasets by SC.

# of top genes	Original data		Permutated data				
	LOOCV errors	*P*-value	0%	25%	50%	75%	100%
50	11	.00	13	17	20	22.75	29
100	11	.00	12	17.25	20	22	30
400	14	.02	13	18	20.5	23	28
1600	13	.00	13	18	20	23	29
6400	13	.02	12	18	20	23	29

**Table 10:** LOOCV two-class misclassification errors with the original Minnesota data and the percentiles of LOOCV errors with 50 permuted datasets by SC: ischemic vs idiopathic.

# of top genes	Original data		Permutated data				
	
	LOOCV errors	*P*-value	0%	25%	50%	75%	100%
50	5	.00	5	10	11	12.75	19
100	5	.00	5	10	11	12	19
400	7	.06	4	9	11	13	17
1600	8	.08	5	9.25	11.5	13	17
6400	9	.12	6	10	11	13	21

#### Simulations

We did a simulation study to further evaluate the performance of the five classification methods. To mimic the real data, simulated data were generated from either a fitted PPLS or a fitted LASSO model to the Minnesota data comparing ischemic vs idiopathic, each containing top 50 genes in the initial model. Specifically, suppose that  is the fitted response value for sample *i *based on the original Minnesota data using PPLS or LASSO. Note that  is a real number without being dichotomized yet. Suppose that *Y_i _*= 1 or -1 is the class label of sample *i *in the original data, and . To generate a simulated data, we independently draw  from Normal distributions  for *i *= 1, ..., 23 and *b *= 1, ..., 50. Then we apply each method to a simulated dataset , where *X*_*i *_is the gene expression profile of sample *i *in the original Minnesota data, and obtain fitted values ; the resulting misclassification error number for dataset *b *is .

Table 11 summarizes the distributions of the misclassification errors of each method based on 50 simulated data with either PPLS or LASSO as the true model. It can be seen that in general all the methods perform similarly, though random forest seems to be most stable and has a slight edge, and the performance of LASSO and nearest shrunken centroid may deteriorate as the number of the genes included in a model is increased. We also did other simulations with the true models starting from various numbers of top genes and various noise levels, and observed similar phenomena: for details see Supplemental Materials.

**Table 11:** Percentiles of misclassification errors from 50 simulated datasets for two-class classification. The true model is either PPLS or LASSO fitted with top 400 genes to the Minnesota data to compare ischemic vs idiopathic.

Methods	# of top genes	True model: PPLS						True model: LASSO					
		
		Mean	0%	25%	50%	75%	100%	Mean	0%	25%	50%	75%	100%
PPLS	50	2.54	0	2	2	3	6	0	0	0	0	0	0
	100	2.6	1	1.25	2	3	7	1	1	1	1	1	1
	400	2.58	0	2	2	3	7	0	0	0	0	0	0
	1600	2.62	0	2	2	3.75	7	0	0	0	0	0	0
	6400	2.6	0	2	2	3	6	0	0	0	0	0	0

PLS	50	2.58	0	2	2	3	6	0	0	0	0	0	0
	100	2.6	0	2	2	3	7	0	0	0	0	0	0
	400	2.56	0	2	2	3	7	0	0	0	0	0	0
	1600	2.52	0	2	2	3	7	0	0	0	0	0	0
	6400	2.54	0	2	2	3	6	0	0	0	0	0	0

LASSO	50	2.48	0	2	2	3	6	0	0	0	0	0	0
	100	2.48	0	2	2	3	6	0	0	0	0	0	0
	400	2.84	0	2	2	3	10	0	0	0	0	0	0
	1600	3.48	0	2	2	4.75	10	0	0	0	0	0	0
	6400	3.48	0	2	2	4.75	10	0	0	0	0	0	0

SC	50	2.76	1	2	2	3	7	1	1	1	1	1	1
	100	2.74	1	2	2	3	7	1	1	1	1	1	1
	400	2.96	0	2	3	3.75	7	0.28	0	0	0	1	1
	1600	3.58	0	2	3	5	8	0	0	0	0	0	0
	6400	4.08	1	3	4	5	10	1	1	1	1	1	1

RF	50	2.48	0	2	2	3	6	0	0	0	0	0	0
	100	2.48	0	2	2	3	6	0	0	0	0	0	0
	400	2.48	0	2	2	3	6	0	0	0	0	0	0
	1600	2.48	0	2	2	3	6	0	0	0	0	0	0
	6400	2.48	0	2	2	3	6	0	0	0	0	0	0

## Discussion

With more and more statistical methods being proposed for discriminant analysis for gene expression data, it has become increasing important to compare and evaluate their performances with real data, as it has been done in other contexts [27]. Comparing the five new methods with each other using the two real datasets, we did not find anyone uniformly better than the others. This may be disappointing to someone who wishes to find the best statistical method. However, in the current application, the similar performance of all the five methods on each of the two datasets provides reassurance on the interesting observation that it is not equally easy to distinguish the different etiologies of heart failure using expression profiles in the two datasets.

Both the *one-against-others *approach and the *pair-wise *approach have been widely used in extending a binary classifier to multi-class settings. Our result suggests that, at least for the two datasets used here, the *one-against-others *approach is better, which was found to be true with support vector machines but in general should also depend on which binary classifier is used [28]. We also have observed that any of the five methods may be sensitive to the number of genes being included. This is particularly relevant because, although all the five methods (and many other methods) can handle any large number of genes, this does not dismiss the potential importance of a user's preliminary ranking and screening of genes. Of course, all our observations here are based on the two datasets without consideration of statistical variability, further studies are needed to validate these points.

An interesting finding of this work is that it is difficult to discriminate the different etiologies of human heart failure using one gene expression dataset, and at the same time, it is quite easy for the other dataset. A possible explanation is the different types of the microarray chips used: Affymetrix HG-U133A chips were used in the Minnesota study while Affymetrix HG-U133 plus 2 chips were used in the PGA study. Because the HG-U133 plus 2 chips contain more genes (or ESTs), to minimize the effects of using different genes, we only used the genes present in the Minnesota data and still yielded much better performance for the PGA data. In fact, we used all the genes in the PGA data and obtained similar results for the PGA data. Although we can say that the performance difference in the two datasets is not caused by different genes contained on a chip, we do not know whether the more recent HG-U133 plus 2 chips provide more reliable measurements on gene expression. In addition, quality control criteria for the inclusion of a chip were nearly identical between the two datasets. We would suspect that the performance difference may be the result of different patient populations and different study protocols (e.g. lack of clearly pre-specified patient inclusion/exclusion criteria). As discussed in [29], a key to validating any prognostic and diagnostic biomarkers is the use of data that can reflect the full range of clinical variability. This highlights the importance of utilizing multiple datasets drawn from multiple subpopulations. Even for the purpose of prediction for one subpopulation, it is possible to improve the performance by borrowing information from other subpopulations [30]. It can be argued that the performance should be weighted on the complexity of the disease. Challenges with the current clinical discrimination of ischemic versus non-ischemic heart failure is indeed why defining potential gene expression biomarkers may be a helpful additional approach in this characterization. A recognized limitation of utilizing heart tissue to identify biomarkers is the difficulty of collecting tissue. In summary, the current and other studies stress the importance of collaborating efforts to share tissue/data to strengthen the search for applicable biomarkers.

## Conclusions

Many studies have aimed to develop new statistical and machine learning methods for best sample discrimination. Our results suggest that, at least for some gene expression data, several existing methods may work almost equally well. More importantly, because of the quite different performances of the methods on the two datasets, one must remain cautious when assessing the performance of sample discrimination using a small gene expression dataset; it may be necessary to use larger or multiple datasets to draw a more reliable conclusion.

## Methods

### Binary classifiers: PLS, PPLS and LASSO

We first briefly review the three binary classifiers, which was first designed for regression and can be directly applied to two-class classification, even when the number of covariates (i.e. genes here) is much larger than the sample size.

We code the response variable (i.e. class label) as *Y *= 1 for class 1 and *Y *= -1 for class 2. Suppose that *x*_*i *_is the expression level of gene *i*, *i *= 1, ..., *p *with *p *as the total number of the genes, and that we have *n *samples in the training data. A challenge is that we have *n *<<*p*.

The main idea of partial least squares (PLS) [21] is to seek a few linear combinations of  for *j *= 1, ..., *m*, then apply ordinary least squares (OLS) to regress *Y *on *z*_*j*_'s to obtain



with β's as OLS estimates. The key of course is how to form linear components *z*_*j*_'s. It turns out that

α_*j *_= argmax_α_Corr^2^(*y*, *Xα*) Var*(Xα)*

with the constraints ||α|| = 1,  for *l *= 1,..., *j *- 1, where *y *is the vector of observed *Y*'s (in the training data), *X *is the design matrix (i.e. matrix of observed *x*'s), and *S *is the sample covariance matrix of *x*'s [31]. In practice, the number of linear components *m *has to be chosen, typically by a form of cross-validation, such as leave-one-out cross-validation (LOOCV), to minimize misclassification errors.

PPLS is a penalized regression method in the framework of PLS [19,32]. Suppose that we have built a PLS linear model, which can be rewritten as:



Then we shrink the PLS coefficients by soft-thresholding [33,34]



where *sign*(*a*) = 1 if *a *≤ 0 and *sign*(*-a*) = -1 if *a *< 0, λ is a shrinkage parameter to be determined, and *f*_+ _= *max*(*f*, 0). It is common that the shrinkage leads to many , effectively eliminating gene *i *from the model, thus gene selection is automatically accomplished. Next we construct a linear component . Finally a PPLS model is built by regressing *Y *on *z *using OLS



which can be re-expressed as . The parameters involved in building a PPLS model, such as the shrinkage parameter λ and the number of PLS components, are estimated by LOOCV. The goal is to choose the largest shrinkage parameter and the smallest number of PLS components for which the LOOCV misclassification error estimate is minimized.

The LASSO estimates [23] in a linear model



are obtained by



subject to , where *Y*_*i *_is the observed response for sample *i *and  is its LASSO estimate, *i *= 1, ..., *n*, and *t *can be chosen by LOOCV. The constraint can often force many , leading to gene selection.

Note that the class label (1/-1) for the response *Y *is binary, but in any of the above binary classifiers, the response *Y *is treated as a continuous variable and the estimate  could be any real number. To predict the class of a new sample, we use *sign*(): if the estimated response  is greater than or equal to 0, then we classify it into class 1; otherwise, class 2. In particular, this direct use of PLS for binary classification (as in [35]) is different from other approaches [36-40]; a distinct advantage of our approach is its simplicity, e.g., avoiding convergence problems when two classes are perfectly separable, which is common in microarray data with a small sample size and a large number of genes.

### Multiclass classifiers: nearest shrunken centroids and random forest

Nearest shrunken centroids (SC) is built on a diagonalized linear discriminant analysis (DLDA) [26,41]. Suppose that we have *K *classes,  is the mean expression level of gene *i *in class *k *of the training samples,  is the pooled sample variance of gene *i *of the training samples, and π_*k *_is the prior probability of a new sample being in class *k*. The DLDA rule for a new sample  is



SC is motivated from the observation that many of the genes will not be predictive of the class membership and should be eliminated from the above DLDA rule. Formally, define



where *n*_*k *_is the number of training samples in class *k*, and  is the overall mean expression level of gene *i *in all the training samples. Note that by the definition, we have . Let



for all *i *and *k*, where Δ is the shrinkage parameter to be chosen by LOOCV. Then substituting  in the DLDA rule by , we obtain a SC rule



The new sample x* is assigned to class *k*_0 _such that .

Note that, if , then  and thus gene *i *plays no role in classifying for class *k*. Hence SC effectively accomplishes gene selection by shrinkage.

Random forest (RF) [24] is an ensemble of classification trees [42,43], which have been shown to be useful in tumor classification with microarray data [44]. It is designed to improve over a single classification tree. There are two random aspects that help generate multiple classification trees in RF. First, a bootstrap sample is repeatedly drawn from the original training data and then used to build a classification tree. Second, in building a classification tree, rather than using the best splitting variable (i.e. gene here) from all the available variables at each node, it chooses the best from a small random subset of all the variables. Each tree is grown to the maximum and no pruning is pursued. To predict the class for a new sample, the sample is applied to each tree and each tree votes by giving its prediction, then the majority vote is taken as the final prediction for the sample.

### Extending a binary classifier to multiclass classification

Here we describe how a multi-class (*K *> 2) classification problem can be handled by a binary classifier, such as PLS, PPLS and LASSO. It is achieved by formulating a multi-class classification problem as multiple two-class classification problems. We consider two most popular approaches: one is to compare each class against all the others, and the other is to compare all possible pairs of classes. Applications of these two approaches can be found, among others, in [45-50]. In particular, some have considered the first approach for PLS [50].

The *one-against-others *approach is to reduce a *K*-class classification task to *K *two-class classification problems. Formally, a new response is defined in the *k*_*th *_binary problem as:



for *k *= 1, ..., *K*. Then we build *K *binary classifiers. To predict a new sample with gene expression profile x*, we apply x* to each binary classifier and yield . Finally, the class of the new sample *C*(x*) is predicted as



That is, the new sample is classified into the class maximizing .

The *pair-wise *approach reduces a *K*-class classification to *K*(*K *- 1)/2 two-class classification problems [45]. Specifically, for each of all possible pairs of classes, solve each of the two-class problems and then, for a new sample, combine all the pairwise decisions to form a K-class decision. Suppose that the new binary response in a pairwise comparison with classes *k*_1 _and *k*_2 _(with 1 ≤ *k*_1 _<*k*_2 _≤ *K*) is defined as



We build a binary classifier with response  using only samples belonging to class *k*_1 _or *k*_2_, and denote the fitted response value for a new sample with expression profile x* as . As described earlier, we classify the new sample into class *k*_1 _or *k*_2 _according to the sign of . After this is done for any 1 ≤ *k*_1 _<*k*_2 _≤ *K*, the final decision is to assign the new sample to the class that wins the most pairwise comparisons. In the case when there are multiple winning classes, we randomly pick one of the winning classes to be the final winning class. Comparing to the *one-against-others *approach, the *pair-wise *approach is computationally more expensive if *K *≥ 4.

### Gene ranking

To explore the effect of the number of genes a model starts with on the classification performance, we have a preliminary gene ranking using a usual F-statistic. This univariate ranking is used throughout, and obviously is by no means to be optimal. For the purpose of the presentation in this section, we only need to consider a given gene. Suppose *x*_*ik *_is the gene expression level of the gene in sample *i *that is in class *k*, *i *= 1, ..., *n*_*k*_, and *k *= 1, ..., *K*, where *n*_*k *_is the total number of samples in class *k *and *K *is the total number of classes. Let  be the mean expression level of class-*k *samples,  be the overall mean (across all the samples) and  be the total number of samples. We can construct an F-statistic as the ratio of the mean sums of squares for between-class and within-class variations:



We can rank all the genes based on their corresponding *F*-statistics: a gene with a larger F-statistic indicates a stronger relationship between its expression levels and the class membership in the samples, and therefore has a higher rank as a potential predictor of the class. We started with various models by including different numbers of top ranked genes. We considered models starting from the top 50, 100, 200, 400, 800, 1600, 3200, 6400, 9200, 12800, 16000, 19200, and all (22283 and 22277 for the two datasets respectively) genes respectively.

It is an incorrect practice in microarray experiments to first select genes using all the samples and then perform cross-validation using the selected genes, which gives downward biased prediction error estimates [51,52]. Hence, it is essential to perform cross-validation on the entire model building process, including gene selection. In our study, we did honest cross-validation. In particular, we cross-validated gene selection (and other aspects of model building, such as parameter selection and estimation). Specifically, in LOOCV, we remove each sample from the data in turn (which is then treated as the test sample), carry out gene selection using F-statistic based on the remaining samples, build a classifier with the selected genes using the remaining samples, and then test the classifier on the left-out sample.

### Data preprocessing

To facilitate the application of penalized regression (i.e. PPLS and LASSO) so that their regression coefficients are in the same unit and thus can be penalized using a global penalty parameter, the expression levels of each gene were scaled to have sample variance 1.

### Evaluations

In addition to PLS/PPLS, we will consider the shrunken centroids (SC) method, the LASSO, and the random forest (RF). SC, LASSO and RF have been implemented in R [53], and are easy to use; we applied their R functions using default parameter settings. SC and RF are directly applicable to multiclass classification while LASSO, as PLS/PPLS, is itself a binary classifier. For multi-class classification with PLS and LASSO, we used the same approaches as described for PPLS.

We use the leave-one-out cross-validation (LOOCV) to estimate the prediction error for each of the methods. Within this first-level LOOCV, a second-level LOOCV is used to select tuning parameters for each method to minimize cross validation errors. Specifically, in PLS, the smallest number of PLS components is selected among the PLS models that give the minimum LOOCV error. In PPLS, among the models with minimum LOOCV error, we first pick the ones with the smallest number of PLS components, then pick the one with the largest shrinkage parameter. In the SC method, the largest shrinkage is selected among the models that minimize the LOOCV error. The number of candidate threshold values and the number of cross validation folds are both set to be default (i.e. 30 and the smallest class size respectively). In LASSO, the maximum fraction parameter of the models that minimize LOOCV error is selected while the number of the candidate fraction values is set to be 51 (equally spaced from 0 to 1) and the number of cross validation folds is set to be the total sample size. In RF, every parameter is set to be default. For example, the number of trees to grow is set to 500, and the number of candidate splitting variables considered at each split is set to  by default, where *p *is the total number of variables (i.e. genes).

Due to the small sample size (about 10 in each class) in each dataset, it is quite challenging to estimate the prediction error well. Although it is straightforward to apply LOOCV or other cross-validation methods, their performance may not be optimal. After submitting this work, we became aware of the recent work by Fu et al [54], where a better method than LOOCV was proposed specifically for microarray data. This new method aims to reduce the variability of LOOCV. We reason that with the use of this new method, the main conclusions drawn in this work would not change.

## Authors' contributions

XH downloaded the PGA data, did the analysis and simulations. WP conceived of and directed the study. SG cleaned and managed the Minnesota data. XH, YC, SJP, LWM and JH conducted the Minnesota study and generated the data. XH, WP and JH drafted the manuscript.

## Supplementary Material

Additional File 1 PLS plots with the first two PLS components. PLS plot for the Minnesota data comparing is-chemic vs idiopathic groups. Click here for file

Additional File 2 PLS plots with the first two PLS components. PLS plot for the PGA data comparing ischemic vs idiopathic groups. Click here for file

Additional File 3 PLS plots with the first two PLS components. PLS plots for LOOCV for the Minnesota data comparing ischemic vs idiopathic groups. Click here for file

Additional File 4 PLS plots with the first two PLS components. PLS plots for LOOCV for the PGA data comparing ischemic vs idiopathic groups. Click here for file

Additional File 5 Simulation results with various simulation set-ups. Click here for file
